# A Multi-Tissue Transcriptomic Atlas of River Buffalo with a Focus on the Genetic Underpinnings of Lactation Performance Across Four Lactation Stages in the Mammary Gland

**DOI:** 10.3390/ijms27094032

**Published:** 2026-04-30

**Authors:** Xinhui Song, Dong Wang, Xier Luo, Chaobin Qin, Ling Li, Yanyan Yang, Yifei Pi, Yanfei Deng, Kuiqing Cui, Zhipeng Li, Wei Xu, Qingyou Liu

**Affiliations:** 1 State Key Laboratory for Conservation and Utilization of Subtropical Agro-Bioresources, College of Animal Science and Technology, Guangxi University, Nanning 530004, China; songxinhui@st.gxu.edu.cn (X.S.); wangdong20201214@163.com (D.W.); 2018401005@st.gxu.edu.cn (C.Q.); lling2010@163.com (L.L.); 2218401005@st.gxu.edu.cn (Y.Y.); yanfei-dun@163.com (Y.D.); zp.li@gxu.edu.cn (Z.L.); 2 Guangdong Provincial Key Laboratory of Animal Molecular Design and Precise Breeding, College of Animal Science and Technology, Foshan University, Foshan 528000, China; luoxier@fosu.edu.cn (X.L.); kqcui@fosu.edu.cn (K.C.); 3 State Key Laboratory of Genome and Multi-Omics Technologies, Shenzhen Branch, Guangdong Laboratory for Lingnan Modern Agriculture, Genome Analysis Laboratory of the Ministry of Agriculture and Rural Affairs, Agricultural Genomics Institute at Shenzhen, Chinese Academy of Agricultural Sciences, Shenzhen 518100, China; piyifei0822@163.com; 4 College of Animal Science, South China Agricultural University, Guangzhou 510642, China

**Keywords:** river buffalo, gene expression atlas, housekeeping genes (HKGs), reference genes, transcriptome, lactation traits

## Abstract

The river buffalo is an economically important livestock species supplying milk and meat. However, a multi-tissue transcriptomic atlas for the key dairy river buffalo breeds, Murrah and Nili-Ravi, has not yet been established, and the lack of stable reference genes has hindered in-depth studies of their biological functions and the molecular mechanisms underlying key economic traits such as lactation. We established a multi-tissue gene expression atlas across 20 tissues and identified 717 housekeeping genes (HKGs), and *RPL37A* and *EEF2* were further shown to be stable candidate reference genes under the conditions tested. We found 8368 tissue-specific genes (TSGs), predominantly enriched in the reproductive system. Exploratory analysis of mammary tissue (dry-period vs. public lactating samples, confounded by batch effects) revealed mammary-enriched hub genes including *LALBA*; these findings are preliminary and require validation. Dynamic analysis across lactation stages (early, peak, mid-, late) identified candidate genes including *SEC14L2* and *ACSM3*. Phenotypic data showed strong negative correlations between milk yield and protein/fat content, and a positive correlation with lactose content. However, causal or regulatory roles were not inferred due to lack of paired individual-level data. Cross-dataset comparisons are descriptive only, and are not key conclusions. In summary, this study lays the foundation for advancing research in lactation trait genetics and functional genomics in river buffalo, with novel reference genes and lactation stage-specific transcriptional dynamics as its main contributions.

## 1. Introduction

The water buffalo (*Bubalus bubalis*) plays an indispensable role in providing nearly 2 billion people worldwide with dairy and meat products, as well as draft animal power. Compared to Holstein cow milk, buffalo milk possesses lower cholesterol content and higher levels of protein, unsaturated fatty acids, calcium, phosphorus, and vitamins [1]. Statistical data from 2025 indicate that the global buffalo population stands at approximately 205 million [2]. Two kinds of water buffalo are classified, including swamp buffalo and river buffalo. River buffalo are primarily distributed in countries in Southeast Asia and along the Mediterranean coast. Among the various dairy river buffalo breeds, the Murrah, Nili-Ravi and Mediterranean buffalo are particularly noted for their excellent lactation performance. As the key organ for milk synthesis and secretion, the mammary gland directly affects milk yield and composition through its structure and function [3]. During lactation, the mammary gland undergoes substantial structural remodeling and functional adaptation, with lactogenic activity being tightly regulated in a time-dependent manner. This results in marked differences in milk production and composition across different stages [4]. Therefore, elucidating the lactational biology and regulatory mechanisms of the mammary gland is crucial for further unlocking the lactation potential of river buffaloes and enhancing milk quality and industry profitability.

Housekeeping genes serve as fundamental safeguards for cellular life activities, maintaining stable expression across various tissues and developmental stages to support essential cellular functions such as basic metabolism, substance transport, and cell cycle regulation [5]. Due to their expression stability, housekeeping genes are commonly utilized as reference genes to normalize variations among samples in quantitative gene expression studies. However, widely used classical reference genes (e.g., *ACTB*, *GAPDH*) exhibit significant limitations, as their expression is not constant across all biological species or tissue types [6]. To address this challenge, researchers have increasingly turned to high-throughput transcriptomic data for the systematic screening and identification of novel, more reliable housekeeping genes. In a study on ducks, a refined panel of 13 highly stable housekeeping genes was identified, including *EEF2*, *RPL6*, and *RPL24*. These genes exhibited exceptionally low expression variability across 50 different tissues, demonstrating superior cross-tissue stability compared with conventional reference genes [7]. In our study on black goats, six key housekeeping genes were identified: *EEF1A1*, *RPS6*, *RPL7*, *RPL23*, *RPS15*, and *RPL19*. Compared with commonly used reference genes in black goats (e.g., *ACTB*, *UBC*, *BCBP3*, *SDHA*), these six genes showed higher and more stable expression levels [8]. By analyzing large-scale expression profiles from multiple tissues and developmental stages, and employing robust bioinformatics algorithms (BestKeeper, Normfinder, geNorm, and RefFinder) to evaluate expression stability, it is possible to identify new candidate reference genes with high expression stability under specific biological systems or experimental conditions [9,10]. The selection of stable reference genes is critical for qPCR normalization, as many conventional genes show developmental instability. Bose et al. identified *RPL32*, *RPL37A*, *HYAL2*, *ACTB*, and *GAPDH* as the most stable reference genes in sheep hearts. Notably, the combination of *HYAL2*, *RPL32*, and *RPL37A* remained unchanged across five developmental stages (fetal to adult), providing an ideal reference gene set for cross-developmental studies [11].

In contrast to HKGs, tissue-specific genes are characterized by their highly restricted expression in particular tissues or organs, where they perform specialized functions relevant to the unique roles of those tissues. Research on tissue-specific genes is of considerable theoretical and applied value, contributing to the in-depth understanding of distinct organ functions, revealing evolutionary and adaptive mechanisms, identifying disease-associated genes and diagnostic biomarkers [12], and screening candidate genes linked to important economic traits in livestock. In studying multi-tissue gene expression, Pan et al. analyzed transcriptomes and identified four housekeeping genes (*COX1*, *UBB*, *OAZ1*/*NPFF*) that maintained stable and high-level expression across all tissues in pig. Moreover, 31 particular TSGs (e.g., *PDC*, *FKBP6, STAT2*, and *COL1A1*) were exclusively expressed in several tissues, including endocrine brain (pituitary gland and pineal gland), ovaries, livers, backfat, jejunum, kidneys, lungs, and longissimus dorsi [13]. Previous efforts have established multi-tissue transcriptomic atlases for selected river buffalo breeds, including *Mediterranean*, *Pandharpuri*, and *Bhadawari* [14], as well as integrated datasets across swamp buffalo [15].

RNA sequencing (RNA-seq) technology, which enables comprehensive transcriptomic profiling by characterizing transcripts, quantifying gene expression, and identifying differentially regulated genes in a single experiment, provides a powerful methodological framework for deciphering the molecular mechanisms underlying complex traits in livestock [16]. Based on this technique, a study has systematically analyzed the global gene expression dynamics in the ovine mammary gland during the transition from late pregnancy to lactation, revealing that genes associated with lipid metabolism undergo significant differential expression regulation during this critical period [3]. Suárez-Vega et al. employed RNA-Seq technology to perform comprehensive transcriptome profiling of the lactating mammary gland in two sheep breeds with distinct milk production traits at multiple time points during lactation [17].

In this study, we expanded the transcriptomic landscape of river buffalo by integrating RNA-seq data from 81 samples across 20 tissues, primarily from Murrah and Nili-Ravi buffalo breeds. Unlike previous transcriptomic studies in buffalo, we systematically evaluated the stability of reference genes across multiple tissues in river buffalo and identified several novel reference genes suitable for future research in this species. Furthermore, we collected milk yield and milk composition data from Murrah and Nili-Ravi buffaloes, and integrated dynamic transcriptional changes during lactation with phenotypic lactation data, providing new insights into the molecular regulation of lactation.

## 2. Results

### 2.1. Multi-Tissue Transcriptomic Characterization of River Buffalo

**Figure 1:**
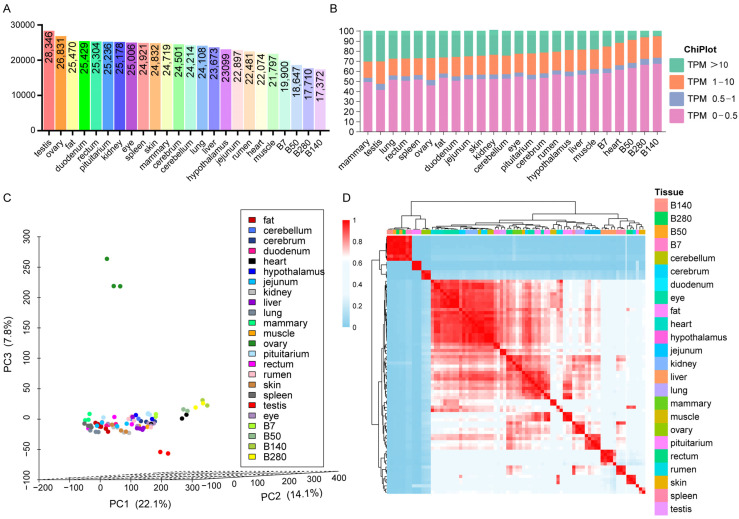
Analysis of gene expression patterns in different tissues of buffalo. (**A**) Bar chart of the number of gene expressions in different tissues; (**B**) bar chart of expression level distribution in different tissues; (**C**) PCA plots of different tissues. PC1: 22.1% variance; PC2: 14.1% variance; PC3: 7.8% variance; (**D**) Pearson correlation heatmap across different tissues.

In total, we analyzed 81 RNA-seq datasets from adult buffalo across 20 major tissues (adipose, cerebellum, duodenum, heart, hypothalamus, jejunum, kidney, liver, lung, mammary gland, muscle, ovary, pituitary gland, rectum, rumen, skin, spleen, testis, eye). We obtained a total of 689.74 Gb of sequencing data, which were processed using fastp (v0.22.0) software to yield 673.96 Gb of high-quality data. The quality-controlled data showed Q20 scores above 95.67% and Q30 scores above 87.82% (Supplementary Table S1). Based on the buffalo NDDB_SH_1 reference genome, a total of 34,781 expressed genes were identified, accounting for 93.58% of the annotated genes. Analysis revealed substantial variation in gene numbers across different tissues, with reproductive tissues demonstrating the most abundant gene expression (testis: 28,346 genes; ovary: 26,831 genes) (Figure 1A). Among other major somatic tissues, the number of expressed genes detected ranged from 25,006 to 21,797.

Notably, the mammary gland exhibited remarkable dynamic changes in gene expression across physiological stages: initiating from the dry period with the highest gene count (24,719 genes), the total number of expressed genes demonstrated a progressive decline during lactation, decreasing to 19,900, 18,647, 17,710, and 17,372 genes during early, peak, late, and mid-lactation stages, respectively (Figure 1A). However, because the dry-period and lactating samples originated from different data sources with complete confounding of batch effects, this observation is exploratory and should be interpreted with caution. This pattern is compatible with the possibility that the mammary gland undergoes progressive changes in gene expression during lactation, potentially associated with stage-specific functional demands. Analysis of the tissue distribution of highly expressed genes (TPM > 10) revealed that the dry-period mammary gland contained the highest proportion of actively transcribed genes, significantly exceeding all other tissues examined. Testicular tissue ranked second in this regard (Figure 1B). To further characterize transcriptomic relationships among samples, we performed principal component analysis (PCA). As shown in Figure 1C, biological replicates of the same tissue clustered together. Notably, testicular and ovarian samples formed distinct clusters clearly separated from somatic tissue samples in the PCA plot, suggesting that the reproductive system may possess a unique gene expression profile distinct from other tissues. Pearson correlation analysis across all samples revealed high mean correlation coefficients (r > 0.9; *p* < 0.001) for the majority of tissues. In particular, mammary gland tissues at different lactation stages (early, peak, mid-, and late) exhibited strong correlations (r > 0.94; *p* < 0.001), consistent with their sharing similar gene expression patterns throughout the lactation cycle (Figure 1D).

### 2.2. Analysis of HKG Expression in River Buffalo

**Figure 2:**
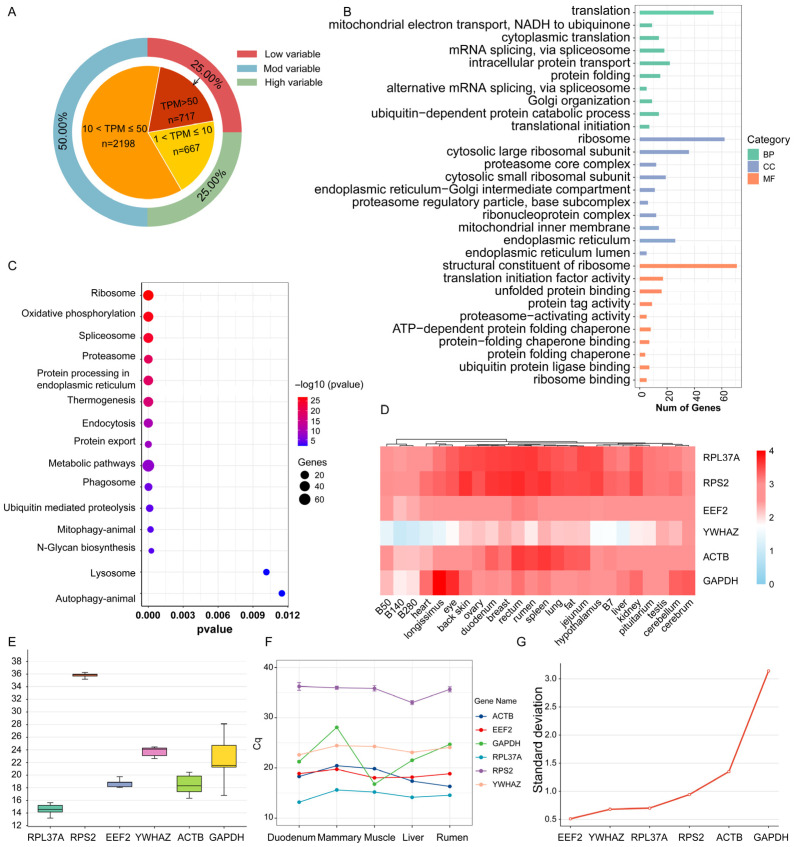
Analysis and validation of HKGs in buffalo. (**A**) Pie–ring chart of preliminary HKGs with different stability and expression level proportions; (**B**) GO enrichment analysis for HKGs; (**C**) KEGG enrichment analysis for HKGs; (**D**) heatmap of candidate and traditional HKG expression; (**E**) boxplot of Cq values for candidate reference genes; (**F**) line plot of BestKeeper analysis; (**G**) line plot of NormFinder analysis.

Genes with mean TPM > 1 were initially considered as candidate HKGs. Analysis revealed that these preliminary HKGs exhibited substantial expression variation across tissues, with the majority demonstrating relatively low expression levels (Supplementary Figure S1A). We classified the initial candidate genes into high- (top 25%), medium- (middle 50%), and low-variability (bottom 25%) groups based on their coefficients of variation across all 20 tissues, and ultimately identified 3582 low-variability genes with stable expression profiles (Figure 2A). Subsequently, this low-variability set was further refined by expression abundance, yielding 717 core HKGs characterized by both low variability and high expression (Figure 2A). To assess the robustness of our HKG selection thresholds, we repeated the screening using more stringent high-expression cutoffs (TPM > 70 and TPM > 100). The candidate reference genes *RPL37A* and *EEF2* were consistently retained across all thresholds (Supplementary Table S2). To validate the functional relevance of this core gene set, we performed GO and KEGG enrichment analyses. Results demonstrated significant enrichment in fundamental cellular processes including translation, protein folding and degradation, mRNA splicing, and intracellular transport. Cellular component analysis revealed enrichment in ribosomes, proteasomes, endoplasmic reticulum, and mitochondrial inner membrane. Molecular function analysis indicated strong associations with ribosomal structure, translation initiation, and protein folding/degradation activities (Figure 2B). Consistently, KEGG pathway analysis showed significant enrichment in core metabolic and cellular maintenance pathways including ribosome, oxidative phosphorylation, spliceosome, proteasome, and protein processing in the endoplasmic reticulum (Figure 2C). The enrichment of low-variability, highly expressed HKGs in these essential cellular pathways confirms their functional validity as HKGs. Further analysis of the top 100 most stable HKGs (by CV value) through heatmap visualization confirmed their high stability and expression levels (Supplementary Figure S1B). From this set, three genes (*RPL37A*, *EEF2*, and *RPS2*) were identified as optimal candidate reference genes due to their exceptional stability and high expression. Comparative analysis demonstrated that these three HKGs exhibited superior expression stability compared to conventionally used reference genes (*ACTB*, *GAPDH*, and *YWHAZ*) (Figure 2D). Furthermore, the stability of the top candidate reference genes (*RPL37A* and *EEF2*) was independently validated by qPCR on five tissues sequenced in our own laboratory, providing within-laboratory confirmation that does not rely on cross-dataset comparisons.

### 2.3. Validation and Selection of Optimal Reference Genes in River Buffalo

To validate the expression stability of candidate reference genes, this study selected three novel HKGs alongside three conventional HKGs for analysis via qPCR. Melting curve analysis demonstrated single peaks for *EEF2*, *YWHAZ*, *RPL37A*, *ACTB*, and *GAPDH*, indicating specific amplification, whereas *RPS2* exhibited anomalous melting curves (Supplementary Figure S2A–F). qPCR expression profiling revealed relatively stable expression across tissues for *EEF2*, *YWHAZ*, *RPL37A*, and *ACTB*; *RPS2* showed low expression levels, while *GAPDH* displayed considerable expression fluctuations (Figure 2E). Further analysis of Cq values demonstrated minimal variation for *RPL37A*, *EEF2*, and *RPS2*, indicating high expression stability. However, *RPS2* exhibited elevated Cq values, reflecting low expression abundance that precludes its suitability as a reference gene. While *YWHAZ* demonstrated satisfactory stability, *ACTB* and *GAPDH* showed poor stability (Figure 2F). For systematic evaluation of gene stability, four algorithms were employed: BestKeeper, Normfinder, geNorm, and RefFinder. BestKeeper and Normfinder analyses yielded identical stability rankings: *EEF2* > *YWHAZ* > *RPL37A* > *RPS2* > *ACTB* > *GAPDH* (Figure 2G; Supplementary Figure S1G). geNorm analysis identified *RPL37A* as the most stable, followed by *YWHAZ*, *EEF2*, *RPS2*, *ACTB*, and *GAPDH* (Supplementary Figure S1H). RefFinder comprehensive evaluation ranked *YWHAZ* as the most stable, followed by *EEF2*, *RPL37A*, *RPS2*, *ACTB*, and *GAPDH* (Supplementary Figure S1I). These analytical results consistently aligned with preliminary qPCR data. In summary, *RPL37A* and *EEF2* demonstrated both high expression abundance across tissues and superior stability compared to *ACTB* and *GAPDH*. Although the conventional reference gene *YWHAZ* exhibited satisfactory stability, its relatively low expression level renders it suboptimal for river buffalo studies. Consequently, this study identifies *RPL37A* and *EEF2* as stable and reliable reference genes for river buffalo tissues.

### 2.4. Identification and Functional Analysis of TSGs Across Multiple River Buffalo Tissues

**Figure 3:**
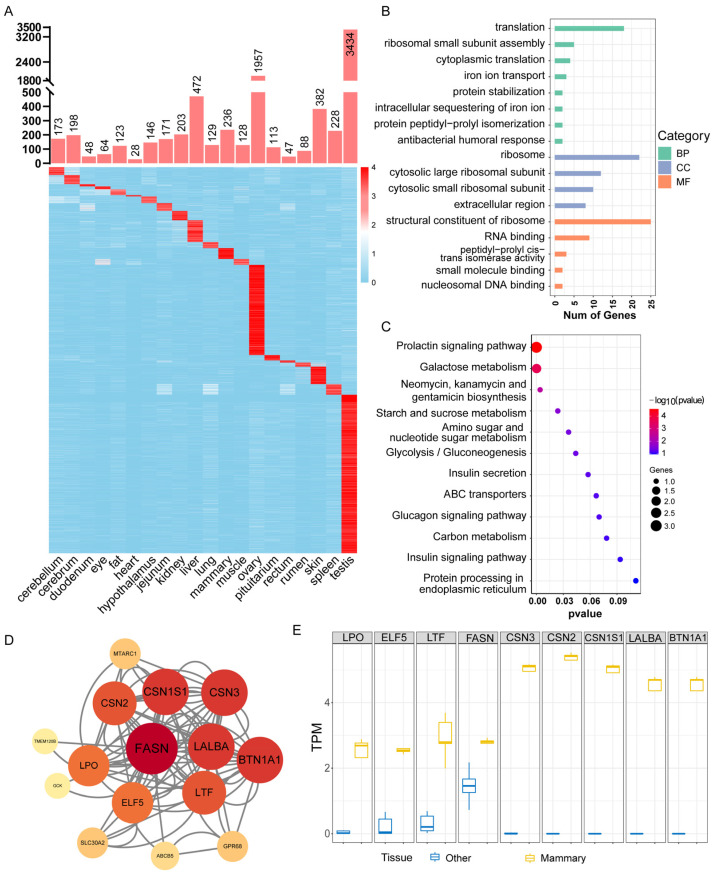
Screening and analysis of TSGs in buffalo. (**A**) Bar plot and heatmap of tissue-specific gene expression; (**B**) GO enrichment analysis for mammary TSGs; (**C**) KEGG pathway enrichment analysis for mammary TSGs; (**D**) PPI network of mammary core genes; (**E**) boxplot of mammary tissue-specific gene expression.

A total of 8368 TSGs were identified through systematic screening. These genes exhibited significant quantitative variation across tissues, with the testis (3434 genes) and ovary (1957 genes) containing substantially more TSGs than other tissues, indicating predominant enrichment in the reproductive system (Figure 3A). Genes exclusively expressed in a single tissue were defined as marker genes. Among the 267 marker genes identified, their distribution pattern closely mirrored that of TSGs, with the testis containing the highest number followed by the ovary. Additionally, mammary gland tissue contained 15 marker genes, while ocular tissue possessed one (Supplementary Table S3). Using DAVID and KOBAS, GO and KEGG analyses were performed on TSGs. The enriched GO terms and KEGG pathways corresponded to the specific functions of the respective tissues. For instance, liver-specific genes were enriched in broad metabolic processes such as xenobiotic metabolism, lipid homeostasis, and cholesterol transport; testis-specific genes were concentrated in reproduction-related events like spermatogenesis, acrosome assembly, and flagellar movement; muscle-specific TSGs mainly involved myofibril assembly, contractile function, and glycogen metabolism. The consistency between the enrichment results and the tissue-specific functions confirmed the credibility of the tissue-specific gene identification process. Eye TSGs were significantly enriched in visual perception, circadian rhythm regulation, and retinoid metabolism, with key genes including *KERA*, *RD3L*, *RDH8*, and *AANAT* demonstrating substantially higher expression in ocular tissue compared to other tissues (Supplementary Figure S3). The mammary TSG list may be confounded by batch effects, and the following mammary-related results should be considered exploratory candidates requiring independent validation. Within this exploratory context, mammary TSGs are primarily enriched in GO functional categories related to translation and ribosomes, pointing to their potential role in regulating protein synthesis (Figure 3B). At the pathway level, they show significant enrichment in the prolactin signaling pathway, galactose metabolism, and glucagon signaling pathway (Figure 3C), suggesting their involvement in hormone regulation and metabolic reprogramming. Protein–protein interaction (PPI) analysis pinpointed nine hub genes within the mammary gland, namely *LALBA*, *BTN1A1*, *CSN2*, *CSN1S1*, *LPO*, *ELF5*, *LTF*, *FASN*, and *CSN3* (Figure 3D). Sensitivity analyses using more stringent fold-change thresholds (4-fold and 5-fold) confirmed that the core mammary hub genes and functional enrichment results were stable across thresholds (Supplementary Table S4). These genes demonstrated substantially elevated expression levels in mammary tissue relative to other tissues. Notably, *LALBA* was further established as a mammary-specific marker gene (Figure 3E).

### 2.5. Dynamic Transcriptional Regulation of the Mammary Gland Across Lactation Stages

**Figure 4:**
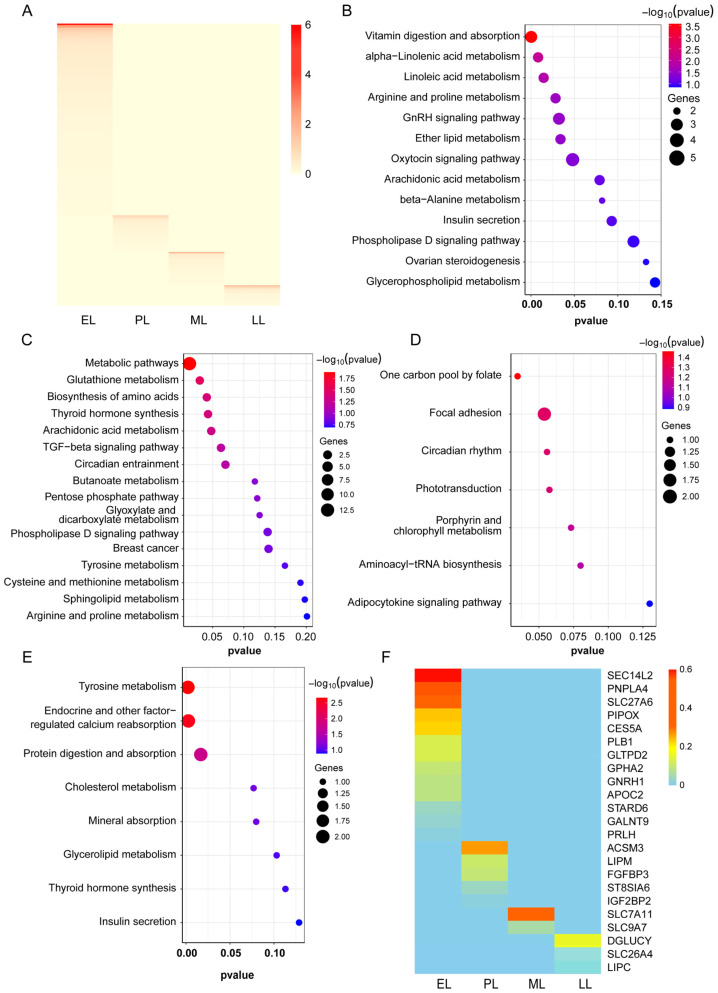
Expression analysis of mammary marker genes across lactation stages. (**A**) Heatmap of mammary marker gene expression across lactation stages (early, peak, mid-, late lactation); (**B**–**E**) KEGG enrichment analyses for mammary marker genes at early, peak, mid-, and late lactation stages; (**F**) heatmap of mammary function-related gene expression.

To elucidate the regulatory mechanisms underlying mammary gland function during the lactation cycle, this study conducted systematic screening and analysis of stage-specific genes across four lactation phases (early, peak, mid-, and late). A total of 2093 stage-specific genes were identified, with their distribution demonstrating dynamic variation: early lactation exhibited the highest transcriptional activity (1220 genes), followed by progressively decreasing numbers during peak (390 genes), mid- (264 genes), and late lactation (219 genes) (Figure 4A). KEGG enrichment analysis revealed that marker genes at each stage were significantly enriched in metabolic and signaling pathways corresponding to their distinct physiological characteristics. Early lactation genes primarily participated in lipid metabolism (α-linolenic acid metabolism, arachidonic acid metabolism), nutrient absorption (vitamin digestion and absorption), and lactation initiation signaling (oxytocin signaling pathway). Peak lactation genes concentrated on fundamental metabolic processes, amino acid biosynthesis, and glutathione metabolism. Mid-lactation genes showed functional shifts toward cell adhesion, circadian rhythm, and folate metabolism. During late lactation, genes were enriched in mineral absorption, cholesterol metabolism, and tyrosine metabolism pathways associated with tissue remodeling (Figure 4B–E). Based on these analyses, we further identified key regulatory genes for each stage, including *SEC14L2* and *PNPLA4* in early lactation, *ACSM3* and *IGF2BP2* in peak lactation, *SLC7A11* in mid-lactation, and *LIPC* in late lactation (Figure 4F).

### 2.6. Variation Patterns of Milk Production and Composition and Their Correlation Analysis

**Figure 5:**
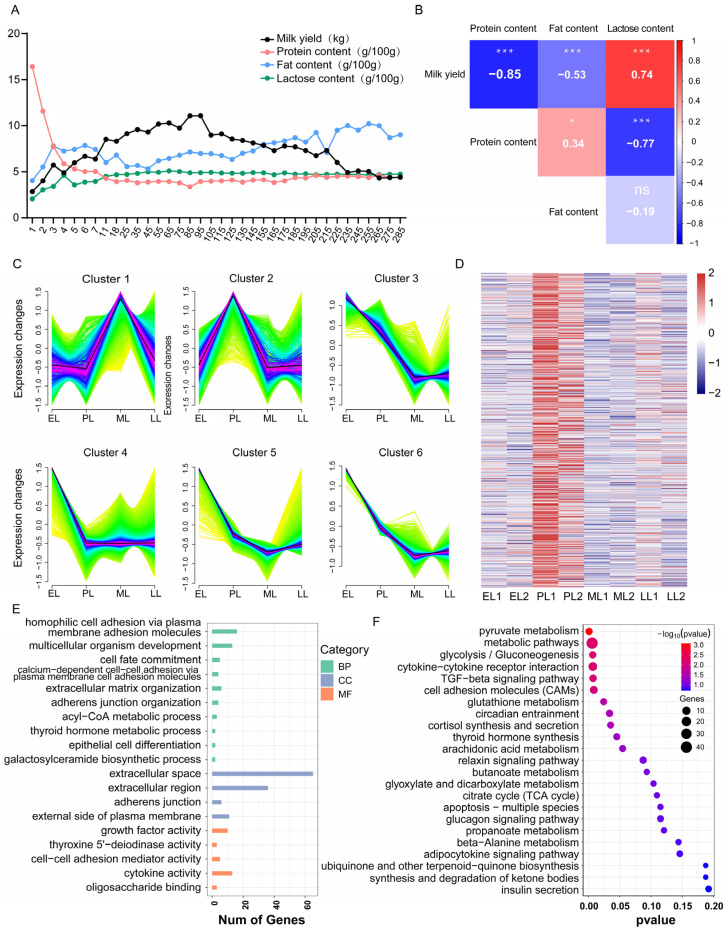
Gene trend analysis of mammary at different lactation stages. (**A**) Changes in the daily average milk yield, protein content, fat content, and lactose content of dairy buffaloes over the entire lactation period. (**B**) Heatmap of correlations between milk yield and composition; * *p* < 0.05, *** *p* < 0.001, and “ns” for non-significant; (**C**) line plot of trend analysis by group; (**D**) heatmap of the gene expression for Cluster 2; (**E**) GO enrichment analysis for Cluster 2 genes; (**F**) KEGG enrichment analysis for Group 2 genes.

This study documented the average lactation data of a mixed population of Murrah and Nili-Ravi buffaloes. Milk yield exhibited a classic lactation curve pattern: a rapid increase during the early lactation stage (days 1–7), followed by a peak plateau period (approximately days 35–115) during which daily milk yield remained high at around 9–11 kg. This was succeeded by a sustained and gradual decline until the late lactation stage (day 285), when yield decreased to 4.42 kg (Figure 5A). Spearman correlation analysis revealed a very strong negative correlation between milk yield and milk protein content (ρ = −0.8508, *p* < 0.001), and a moderate negative correlation with milk fat content (ρ = −0.5295, *p* < 0.001), confirming the presence of a dilution effect (Figure 5B). In contrast, milk yield showed a strong positive correlation with lactose content (ρ = 0.7428, *p* < 0.001). As the primary osmotic regulator, increased lactose synthesis helps maintain osmotic pressure and promotes milk secretion. Protein content exhibited a strong negative correlation with lactose (ρ = −0.7690, *p* < 0.001), which can be attributed not only to the trade-off in solute concentrations under the constraint of milk isotonicity but also suggests potential competition between their synthetic pathways (Figure 5B). A comparative analysis of milk yield and milk composition across different lactation stages (early, peak, mid-, and late lactation) revealed distinct variation patterns along the lactation progression. Specifically, significant differences in milk yield were observed between early and peak lactation, peak and late lactation, and mid- and late lactation. Milk protein and lactose contents exhibited similar variation trends, with both being highly significantly lower in early lactation than in all subsequent stages. In contrast, milk fat content showed a significant difference only between peak and late lactation (Supplementary Figure S4A–D).

### 2.7. Changing Patterns of Gene Expression Dynamics and Lactation Traits Across Lactation Stages

Trend analysis was further performed to cluster all genes, resulting in six clusters with distinct expression patterns. Cluster 2, comprising 979 genes, exhibited an expression trend consistent with the changes in milk yield (Figure 5C). Heatmap analysis showed that the expression levels of these genes were markedly higher during peak lactation compared to other stages, aligning with the trend analysis results (Figure 5D). To elucidate the biological functions of this gene cluster, GO annotation and KEGG pathway enrichment analyses were conducted. GO analysis indicated that these genes were significantly enriched in biological processes related to cell adhesion and developmental regulation (Figure 5E). KEGG pathway analysis further indicated that these genes are widely involved in core energy metabolism pathways (e.g., glycolysis/gluconeogenesis), cell signaling and interaction-related pathways (e.g., TGF-β signaling pathway), and various hormone and lipid metabolism processes (e.g., thyroid hormone synthesis, arachidonic acid metabolism, relaxin signaling pathway) (Figure 5F). Additionally, through trend analysis we further explored the association between gene expression and lactation traits. Based on Cluster 2, which exhibited an expression pattern consistent with changes in milk yield, we identified several genes whose expression dynamics closely mirrored specific lactation traits. For instance, the expression levels of *CCL26*, *LRAT*, *LOC102397154*, and *HAPLN3* increased during peak lactation, corresponding to the peak in milk yield. *LOC102390798* (*FOLR1*) and *RASD1* showed higher expression in early lactation, while their expression relatively decreased as lactose content rose during peak lactation. *GP2* and *LARGE1* displayed elevated expression in late lactation, when milk protein and fat contents remained relatively high but milk yield and lactose content declined. Furthermore, the expression pattern of *CCN2* aligned with changes in milk protein content during mid-to-late lactation, and *LYPD5* expression followed the trend of milk fat content.

## 3. Discussion

With a global population exceeding 200 million and a domestication history spanning 3000–6000 years, river buffaloes are primarily utilized for dairy production. Therefore, screening housekeeping genes (HKGs) across multiple tissues in buffaloes is critical for investigating gene expression regulation in these tissues. HKGs are constitutively expressed across tissues and cell types, maintaining fundamental cellular functions. However, numerous studies have demonstrated that reference gene stability is context-dependent, varying across species, tissues, and physiological states; thus, no single universal reference gene exists for all conditions [18]. This underscores the necessity for systematic identification of optimal HKGs tailored to specific experimental models. In the present study, we performed a comprehensive analysis of gene expression patterns across 20 river buffalo tissues. From an initial set of genes with mean TPM > 1, we rigorously selected 717 core HKGs characterized by both low expression variability and high abundance. Functional enrichment analyses confirmed that these genes are significantly involved in essential cellular processes and structures, such as translation, ribosome biogenesis, oxidative phosphorylation, protein folding/degradation, and intracellular transport. This functional profile aligns with findings in other species (e.g., human, cattle, and pig) [5,13,19], where HKGs consistently converge on fundamental cellular maintenance pathways, despite differences in gene identity. Our results reinforce the evolutionary concept that while the specific repertoire of HKGs may differ among species, their core biological functions are conserved.

Three genes (*RPL37A*, *EEF2,* and *RPS2*) were identified as exhibiting exceptional expression stability across buffalo tissues. Subsequent qPCR validation across multiple tissues, analyzed using established algorithms (BestKeeper, Normfinder, geNorm, and RefFinder), confirmed that *EEF2* and *RPL37A* displayed superior stability and higher expression levels compared to traditionally used reference genes (*ACTB*, *GAPDH*, and *YWHAZ*). This finding is consistent with prior studies in other models: *EEF2* has been validated as a stable reference in goat skin [20] and Ph-like acute lymphoblastic leukemia [21], while *RPL37A* has shown stability in human meningiomas [22], ovarian tissues [23] and sheep heart development [11]. Although *RPS2* demonstrated lower expression abundance in our qPCR assay, making it less ideal as a standalone reference, the combined evidence strongly supports *EEF2* and *RPL37A* as robust reference genes for gene expression normalization in multi-tissue studies of river buffalo. A limitation of this qPCR analysis is the absence of primer efficiency measurements, which affects the accuracy of cross-gene comparisons. Under identical amplification conditions, the comparison of Cq values for the same gene across tissues is less sensitive to, but not independent of, efficiency differences. Without measured efficiencies, any quantitative comparison of Cq values between distinct genes remains tentative. In addition, the HKG identification and analysis presented here do not rely on problematic cross-dataset comparisons, as the stability ranking was derived from the full multi-tissue dataset, and the qPCR data were generated entirely from our own samples.

We identified a total of 8368 TSGs across 20 buffalo tissues, with the testis (3434 genes) and ovary (1957 genes) harboring the highest numbers. This predominant enrichment in the reproductive system aligns with findings in goats and cattle [19], suggesting a conserved pattern of high transcriptional specialization in mammalian gonads. A key finding was the identification of TSGs in the highly specialized ocular tissue. Genes such as *KERA*, *RD3L*, *RDH8*, and *AANAT* were not only specifically expressed but also functionally enriched in visual perception, circadian rhythm, and retinoid metabolism. Mutations in these genes are associated with various visual impairments in other species, including cornea plana (*KERA*) [24] and susceptibility to myopia (*RD3L*) [25]. *RDH8* and *AANAT* play critical roles in phototransduction and melatonin synthesis, respectively, with their dysregulation linked to photoreceptor degeneration and disrupted circadian cycles [26,27,28]. The specific enrichment of these functionally coherent gene sets in buffalo eye tissue validates our screening approach and highlights potential candidate genes for studying visual physiology and disorders in this species.

Our analysis of mammary gland TSGs yielded particularly insightful results. We identified nine hub genes (*LALBA*, *BTN1A1*, *CSN2*, *CSN1S1*, *LPO*, *ELF5*, *LTF*, *FASN*, and *CSN3*) through protein–protein interaction (PPI) network analysis, all showing markedly elevated expression in mammary tissue. These genes form a functional core network governing essential mammary functions: milk protein synthesis (*CSN1S1*, *CSN2*, *CSN3*, *LALBA*), lactogenesis regulation (*ELF5*), lipid synthesis and secretion (*FASN*, *BTN1A1*), and innate immune defense (*LPO*, *LTF*). Their roles in determining milk yield and composition are well-documented [29,30,31,32,33,34]. Notably, we further established *LALBA* as a mammary-specific marker gene. *LALBA* is involved in the synthesis of lactose synthase in the mammary gland, promoting milk production and secretion. It can bind divalent cations such as Ca^2+^ and Zn^2+^ and may facilitate the absorption of essential minerals [29]. In mammary epithelial cells, *LALBA* combines with the membrane-bound β-1,4-galactosyltransferase to form lactose synthase, which catalyzes lactose formation in the Golgi apparatus, thereby regulating milk yield. Since lactose cannot diffuse out of secretory vesicles, water plays a role in maintaining osmotic balance [29]. The co-expression and interaction of these hub genes suggest a tightly coordinated regulatory program underlying buffalo lactational biology. Beyond static tissue specificity, we also explored dynamic changes by identifying 2093 genes specifically expressed during different lactation stages. Key stage-specific genes like *SEC14L2* [35], *SLC27A6* [36], *GNRH1* [37], *APOC2* [16], and *PRLH* [38] in early lactation and *SLC7A11* and *SLC26A4* [39] in mid-to-late lactation have been implicated in lipid metabolism, hormone signaling, and stress response in other dairy species. Their specific expression in the buffalo mammary gland at respective stages underscores their potential role in orchestrating the metabolic and functional reprogramming necessary for successful lactation. However, a caveat applies to the mammary TSG list: because our own mammary samples were all from the dry period and the public mammary samples were all from lactation, the comparison between mammary and other tissues may be confounded by batch effects. Therefore, the mammary TSGs identified in this study should be regarded as exploratory candidates requiring independent validation, and they are not presented as a key conclusion of this work. All interpretations involving dry-period vs. lactating comparisons (including the higher gene count noted in Figure 1A) are similarly exploratory.

The lactation performance of dairy animals is a dynamic process characterized by stage-specific changes in milk yield and composition. In this study, longitudinal analysis of Murrah and Nili-Ravi buffaloes revealed a classical lactation curve and confirmed a strong negative correlation between milk yield and milk protein content (ρ = −0.8508), alongside a positive correlation with lactose (ρ = 0.7428). These correlations are consistent with the well-documented “dilution effect” in dairy ruminants [40,41], where increased milk volume dilutes the concentration of milk solids, while elevated lactose synthesis, as the primary osmotic driver, facilitates higher milk secretion. To decipher the molecular underpinnings of these phenotypic patterns, we performed transcriptomic analysis across four lactation stages. A key finding was the identification of a gene cluster (Cluster 2, 979 genes) whose expression trajectory perfectly mirrored the lactation curve. The enrichment of these genes in pathways related to cell adhesion, developmental regulation, TGF-β signaling, and relaxin signaling provides crucial mechanistic insight [42]. This suggests that beyond basic metabolism, dynamic remodeling of the mammary epithelial structure and cell–cell communication, potentially mediated by hormonal pathways like relaxin, are integral to sustaining peak lactation output.

In this study, we identified multiple genes associated with lactation traits, including *CCL26*, *PRELP*, *GP2*, and *CCN2*. *PRELP* has been implicated in modulating gut microbiota, which may indirectly influence nutrient utilization and health during lactation [43]. *GP2* is a robust biomarker of high lipolysis in cow milk [44]. Research has demonstrated an association between the *FOLR1* gene and milk production traits in goats [45]. Analysis of RNA in milk fat during the transition from bovine colostrum to mature milk revealed that genes whose expression is upregulated in parallel with milk yield encode proteins including α-lactalbumin (*LALBA*), β-casein (*CSN2*), and folate receptor alpha (*FOLR1*) [46]. *CCN2* is a versatile signal modulator that affects a broad range of biological or pathological processes including bone growth, new blood vessel formation, kidney and skin disease, and tumor development [47]. The transcription of *CCN2* is controlled by multiple extracellular stimuli, including factors such as TGF-β, angiotensin II (Ang II), interleukin-1, thrombin, and epidermal growth factors, as well as environmental stimuli such as hypoxia and mechanical tension. These observations merely reflect temporal parallelism between gene expression and lactation traits; because individual-level paired data and statistical association tests are lacking, this study does not infer any causal or regulatory roles.

Based on transcriptomic data, we propose the following hypothetical mechanisms underlying lactation performance: The high expression of *LALBA* during peak lactation may increase milk yield by promoting lactose synthesis, which osmotically drives water secretion into milk—consistent with the positive correlation between lactose content and milk yield (ρ = 0.7428). The upregulation of *SEC14L2* and *ACSM3* during early and peak lactation may support lipid transport and fatty acid activation for milk fat synthesis, and their subsequent decline parallels the natural decrease in milk fat percentage. The negative correlations between milk yield and protein/fat (ρ = −0.8508/–0.5295) and the positive correlation with lactose (ρ = 0.7428) may partly reflect a transcriptional imbalance, where volume-driving genes (e.g., *LALBA*) and protein/fat synthesis genes (e.g., *CSN1S1*, *FASN*) exhibit a relative transcriptional imbalance that may contribute to a dilution effect. Candidate genes such as *CCL26* and *FOLR1* may be involved in mammary cell turnover or one-carbon metabolism, thereby influencing lactation traits. We emphasize that all these proposals are hypothesis-generating and based solely on correlational evidence; experimental validation (e.g., gene perturbation in mammary epithelial cells or animal models) is required to establish causality.

The following limitations should be considered when interpreting the results of this study: The multi-tissue atlas was based on four buffaloes. Due to difficulty in sampling certain tissues (e.g., hypothalamus) and the exclusion of samples with an RNA integrity number (RIN) < 7, the effective number of biological replicates per tissue ranged from two to four (detailed in Supplementary Table S1). Our expression atlas was based on 2–4 biological replicates per tissue, and the public lactation transcriptome had only two replicates per stage. The limited sample size restricts the precision of expression estimates, increases the risk that results are influenced by sampling variation, and may reduce reproducibility. This is particularly concerning for low-abundance genes, where mean expression levels and variability derived from a few samples may not stably represent the population. Consequently, the candidate genes identified in this study should be considered exploratory and require validation in larger, independent cohorts. We have labeled these findings as exploratory and recommend independent validation in larger cohorts. A major limitation is that the comparison between lactating and dry-period mammary gland tissues is confounded by data source: all lactating samples were obtained from a public database (PRJNA480718), while all dry-period mammary samples were generated in our own laboratory. Because batch effects are completely confounded with biological condition (lactating vs. dry), any observed differences cannot be unambiguously attributed to lactation stage alone. Therefore, all interpretations involving cross-dataset comparisons (including the higher gene count in dry-period mammary glands reported in Figure 1A and the mammary TSG list in Section 2.4) are exploratory and require independent validation; these cross-dataset findings are not presented as key results of this study. The lactation phenotype data came from six independent buffaloes. The small sample sizes limit statistical power, and the phenotype data were not matched to the transcriptome samples, potentially introducing batch effects and population differences. This study did not perform functional experiments to validate candidate genes (e.g., *SEC14L2*, *ACSM3*, *CCL26*). Future studies in buffalo mammary epithelial cell lines or animal models are required to test the biological roles of these genes. Because this study includes only four lactation time points (early, peak, mid-, late), the interpretation of dynamic changes should be considered preliminary. Finer temporal resolution (e.g., weekly or monthly sampling) would be required to reveal the complete transcriptional regulatory trajectory. In summary, the main conclusions of this work—namely the identification of stable housekeeping genes (*RPL37A* and *EEF2*) with qPCR validation, and the lactation stage-specific transcriptional dynamics within the public dataset—do not rely on the problematic cross-dataset comparisons and are supported by independent within-dataset or within-laboratory data.

## 4. Materials and Methods

### 4.1. Tissue Sampling

For this experiment, two Murrah buffaloes and two Nili-Ravi buffaloes were sourced from the breeding farm of the Guangxi Zhuang Autonomous Region Buffalo Research Institute, where they were kept under identical feeding and management conditions. The buffaloes were slaughtered via instantaneous high-voltage electrocution, and tissue samples from 18 different organs/tissues (adipose, cerebellum, duodenum, heart, hypothalamus, jejunum, kidney, liver, lung, mammary gland, muscle, pituitary, rectum, rumen, skin, spleen, testis, and eye) were collected from each of the four adult buffaloes. The samples were minced, placed in pre-labeled cryovials, immediately flash-frozen in liquid nitrogen, and stored at −80 °C. The animal experiments were approved by the Animal Experiment Ethics Review Committee of Guangxi University, Nanning, China (Approval No.: Gxu-2021-1006).

### 4.2. Library Construction and Transcriptome Sequencing

Library construction and transcriptome sequencing were outsourced to BGI-Wuhan (BGI Genomics Co., Ltd., Wuhan, China). Total RNA was extracted from the samples using Trizol reagent, genomic DNA was removed using DNase I, and RNA quality and concentration were measured using NanoDrop 2000 (Thermo Fisher Scientific, Wilmington, DE, USA), followed by cDNA synthesis and amplification. The cDNA samples were sonicated using the Bioruptor ^®^ Sonication System (Diagenode Inc., Denville, NJ, USA) to generate small fragments with an average length of 350 bp. Subsequently, end repair, A-tailing, and adapter ligation steps were performed, and magnetic beads (Beckman Ampure XP, Beckman Coulter, Brea, CA, USA) were used for purification after each reaction. The adapter-ligated products were then subjected to PCR amplification. The concentration and fragment distribution of the amplified cDNA products were assessed using a Fragment Analyzer (Agilent Technologies, Santa Clara, CA, USA). Libraries were constructed upon passing quality control checks. Sequencing libraries were generated using the NEB Next Ultra RNA Library Prep Kit for Illumina (New England Biolabs, Ipswich, MA, USA). Sequencing was performed on the DNBSEQ-T7 platform with paired-end 150 bp (PE150) reads, using a non-strand-specific poly-A enrichment library preparation method. Furthermore, this study acquired RNA-seq data of buffalo jejunum, ovary, and mammary gland tissues at different lactation stages from the NCBI SRA database (https://www.ncbi.nlm.nih.gov/sra (accessed on 27 June 2024); Supplementary Table S1). Integrated with the sequencing data from self-collected samples, the dataset comprehensively covers 20 distinct tissue types, comprising 81 RNA-seq samples, including our newly sequenced samples; the clean data volume ranged from approximately 6.99 to 11.83 Gb per sample (as detailed in Supplementary Table S1).

We implemented a standardized analytical workflow for all samples. Initial quality control was performed using fastp (v0.22.0) with default parameters to process raw RNA-seq data into high-quality clean sequences [48]. The resulting data underwent comprehensive evaluation through FastQC (v0.12.1) to verify sequencing quality. For genomic alignment, we constructed the reference index through the hisat2-build utility within HiSAT2 (v2.2.1), utilizing the reference genome sequence as input [49]. Subsequent alignment involved mapping clean reads to the reference genome (NDDB_SH_1) using HiSAT2. The resulting SAM files were converted to BAM format through Samtools (v1.9), with comprehensive alignment statistics generated via samtools stats functionality [50]. Transcript assembly for individual samples was conducted using StringTie (v2.1.7) [51]. We utilized StringTie’s merge function (stringtie -p 20 -e -b) to combine all sample assemblies into a unified transcriptome. Finally, normalized gene expression values were calculated as transcripts per million (TPM) using the same software. We selected genes with TPM > 1 in at least one sample across all samples to generate a PCA clustering plot. Meanwhile, we calculated the average gene expression values within replicate samples (samples from the same tissue) and used these averages for inter-tissue correlation analysis. The Pearson correlation coefficient (r) and the associated *p*-values for testing significance were calculated using the Hmisc (v5.1) package.

### 4.3. Detection of Housekeeping Genes and Expression Stability Evaluation of Candidate Reference Genes

Genes with an average TPM > 1 were preliminarily defined as HKGs, and their expression stability was further evaluated using the coefficient of variation (CV) [52]. Based on the quartiles of the overall distribution, the CV was categorized into a low-variability group (CV ≤ first quartile), medium-variability group (first quartile < CV < third quartile), and high-variability group (CV ≥ third quartile). According to their average expression levels across tissues, the low-variability group was further divided into low-expression (1 < TPM ≤ 10), medium-expression (10 < TPM ≤ 50), and high-expression (TPM > 50) subgroups. Genes with low variability (CV ≤ first quartile) and high expression (TPM > 50) were defined as candidate HKGs [8]. To assess the robustness of housekeeping gene (HKG) selection criteria (mean TPM > 1, CV ≤ first quartile, TPM > 50), we repeated the screening using more stringent high-expression thresholds (TPM > 70 and TPM > 100).

Three candidate reference genes (*RPL37A*, *EEF2*, and *RPS2*) and three commonly used reference genes (*ACTB*, *GAPDH*, and *YWHAZ*) were selected (Supplementary Table S5). cDNA samples from five tissues (mammary gland, rumen, duodenum, liver, and muscle) were extracted, and their RNA concentration and purity were measured. Qualified samples were reverse-transcribed according to the instructions of the GoScript™ Reverse Transcription System kit. Specific primers were designed using Primer 5.0 based on gene sequences obtained from NCBI. The Cq values of each gene in the five tissues were obtained via qPCR. Expression stability was evaluated using three algorithms—geNorm, NormFinder [9], and BestKeeper [53]—and the results were integrated via the RefFinder platform to comprehensively identify the optimal reference gene. The Cq values of the six genes in the five tissues were obtained. The Cq data of all candidate reference genes from the qPCR experiment were evaluated using geNorm, NormFinder, and BestKeeper methods. geNorm calculates the expression stability measure (M) by determining the average pairwise variation of each candidate reference gene, where a lower M value indicates higher stability. NormFinder analyzes the expression stability of genes between and within groups and determines the standard deviation (SD) through advanced analysis. Compared to geNorm and NormFinder, BestKeeper directly uses the Cq values obtained from the software to evaluate the best reference genes, where genes with lower SD values are considered potential reference genes. Finally, RefFinder integrated the results of the three algorithms and evaluated and screened the candidate reference genes by efficiently calculating the geometric mean of the Cq values in the samples [10].

### 4.4. Identification of TSGs and PPI Network Construction

Genes with low expression levels (TPM < 1) across all tissues were initially excluded. The filtered TPM matrix was subsequently normalized using the qsmooth package (v1.4.0) in R [54]. Following normalization, the mean TPM values were calculated for all biological replicates within each tissue. TSGs were defined as those exhibiting expression levels in a given tissue that were threefold higher than in all other tissues [55]. For tissue-specific gene (TSG) identification (original threshold: ≥3-fold change), we tested more stringent thresholds of 4-fold and 5-fold. Furthermore, marker genes were identified as genes exclusively expressed in a single tissue. A protein–protein interaction (PPI) network was constructed for mammary TSGs in dairy buffalo using STRING (v12.0) software, from which significant gene networks were extracted [56]. Subsequently, the topological properties of the PPI network were analyzed and visualized with Cytoscape (v3.10.0) to identify hub genes within the mammary tissue.

### 4.5. Sample Collection and Composition Analysis Across Lactation Stages

Daily milk yield was recorded for six lactating buffaloes (comprising three Murrah and three Nili-Ravi buffaloes). Raw milk samples were collected starting from day 1 of lactation. During the first 25 days, sampling was performed intensively on days 1, 2, 3, 4, 5, 6, 7, 11, 18, and 25; thereafter, samples were collected every 10 days until the end of lactation. Milk yield data from days 7, 85, 145, and 285 were selected to represent the early, peak, mid-, and late lactation stages, respectively. The methodology for milk composition analysis was as follows: Milk protein, fat, and lactose contents were automatically determined in duplicate using a MilkoScan FT120 multifunctional milk analyzer (Foss, Hillerød, Denmark) and averaged. Spearman’s rank correlation coefficient was used to assess the relationships among four variables: milk yield, protein content, fat content, and lactose content. Spearman’s ρ is a non-parametric measure that does not assume normality and is suitable for evaluating monotonic associations. All analyses were performed using R software with the Hmisc (v5.1) package. The rcorr function was applied to the data matrix to obtain the correlation coefficients and their corresponding *p*-values. Correlations with *p* < 0.05 were considered statistically significant.

### 4.6. Trend Analysis of Mammary Gland Across Lactation Stages

The Mfuzz (v2.46.0) package, which employs fuzzy c-means clustering, was utilized to group genes with similar expression patterns into distinct clusters [57]. Subsequently, trend analysis of mammary gland genes across different lactation stages was performed using Mfuzz, with trend diagrams generated to visualize expression dynamics. Genes demonstrating expression trends correlated with milk yield were selected for heatmap visualization.

### 4.7. Functional Enrichment Analysis

Functional enrichment analysis was performed using DAVID for Gene Ontology (GO) terms [58] and KOBAS for KEGG pathways [59]. The results were visualized using the ggplot2 package (v3.4.2) in R software (v4.2.3).

## 5. Conclusions

In this study, we constructed a multi-tissue transcriptomic atlas of river buffalo, primarily based on Murrah and Nili-Ravi breeds, using RNA-seq data from 20 major tissues. We identified a core set of 717 housekeeping genes and validated *RPL37A* and *EEF2* as novel stable reference genes using multiple algorithms and qPCR. Systematic screening revealed 8368 tissue-specific genes, with *LALBA* confirmed as a mammary-specific marker. Dynamic analysis across four lactation stages revealed stage-specific transcriptional programs and key regulatory genes, such as *SEC14L2* and *ACSM3*. Milk production data exhibited a classic lactation curve. Integrated trend analysis identified a gene cluster (Cluster 2) whose expression pattern paralleled milk yield changes, and recognized candidate genes including *CCL26*, *FOLR1*, and *GP2* associated with specific lactation traits. Together, this study provides a useful resource for functional annotation of the buffalo genome and future investigations into the molecular regulation of lactation.

## Data Availability

The raw sequencing data have been deposited in the NCBI Sequence Read Archive (SRA) under BioProject accession PRJNA1429809 (https://www.ncbi.nlm.nih.gov/bioproject/PRJNA1429809, accessed on 17 March 2025).

## Supplementary Materials

The following supporting information can be downloaded at https://www.mdpi.com/article/10.3390/ijms27094032/s1.

## Abbreviations

HKGs	Housekeeping Genes
TSGs	Tissue-specific Genes
GO	Gene Ontology
KEGG	Kyoto Encyclopedia of Genes and Genomes
PCA	Principal Component Analysis
