# Supplementary material for: A Multi-Tissue Transcriptomic Atlas of River Buffalo with a Focus on the Genetic Underpinnings of Lactation Performance Across Four Lactation Stages in the Mammary Gland
Source: Int J Mol Sci. 2026 Apr 30;27(9):4032. doi: 10.3390/ijms27094032 (PMC13163570; doi:10.3390/ijms27094032)
Supplement: Supplementary file 1 [file ijms-27-04032-s001.zip › ijms-4248864-supplementary.pdf]

**SUPPLEMENTARY INFORMATION**

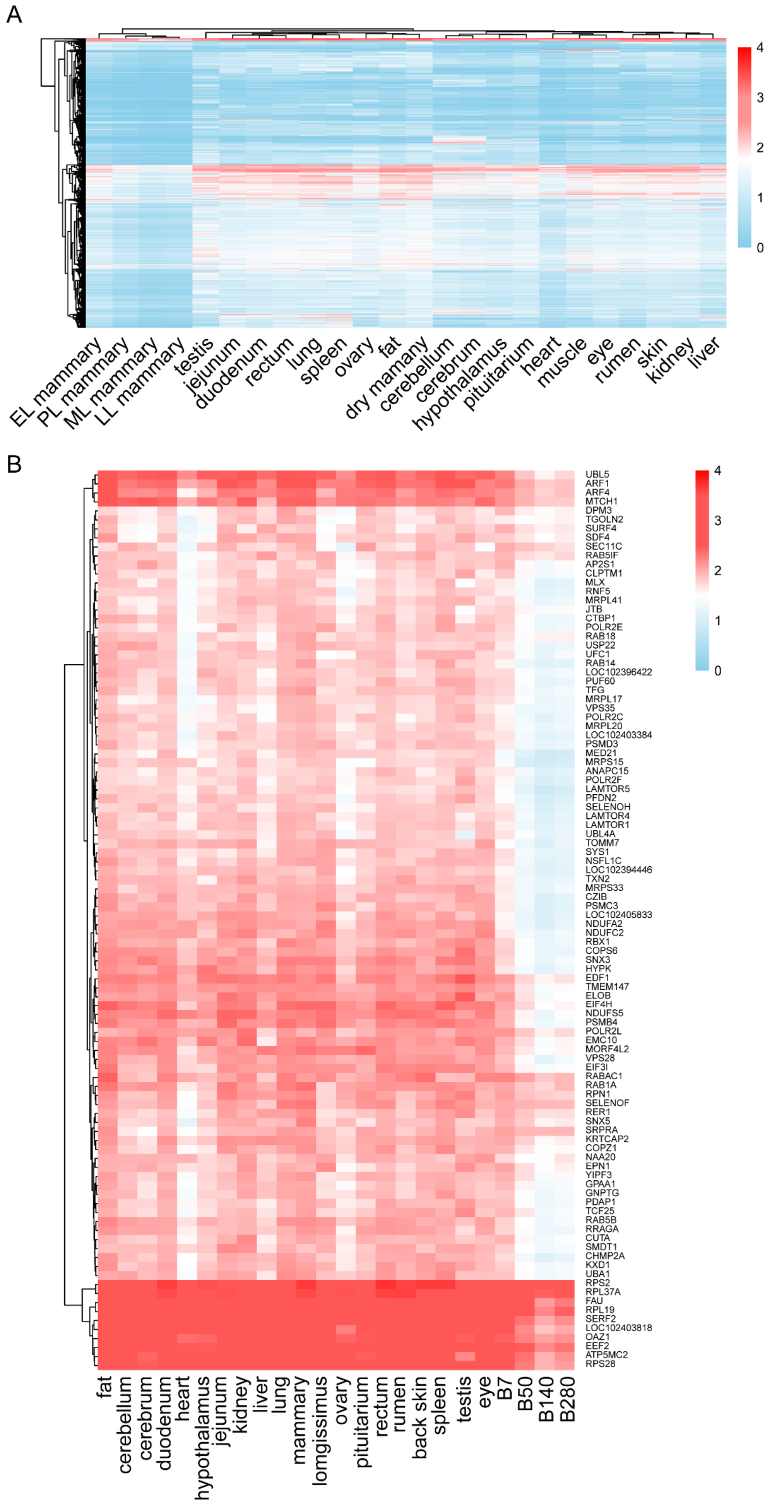


**Supplementary Figure S1 Screening of HKGs in buffalo.** (A) Heatmap of preliminary housekeeping gene expression; (B) Heatmap of expression levels for the top 100 most stable HKGs.

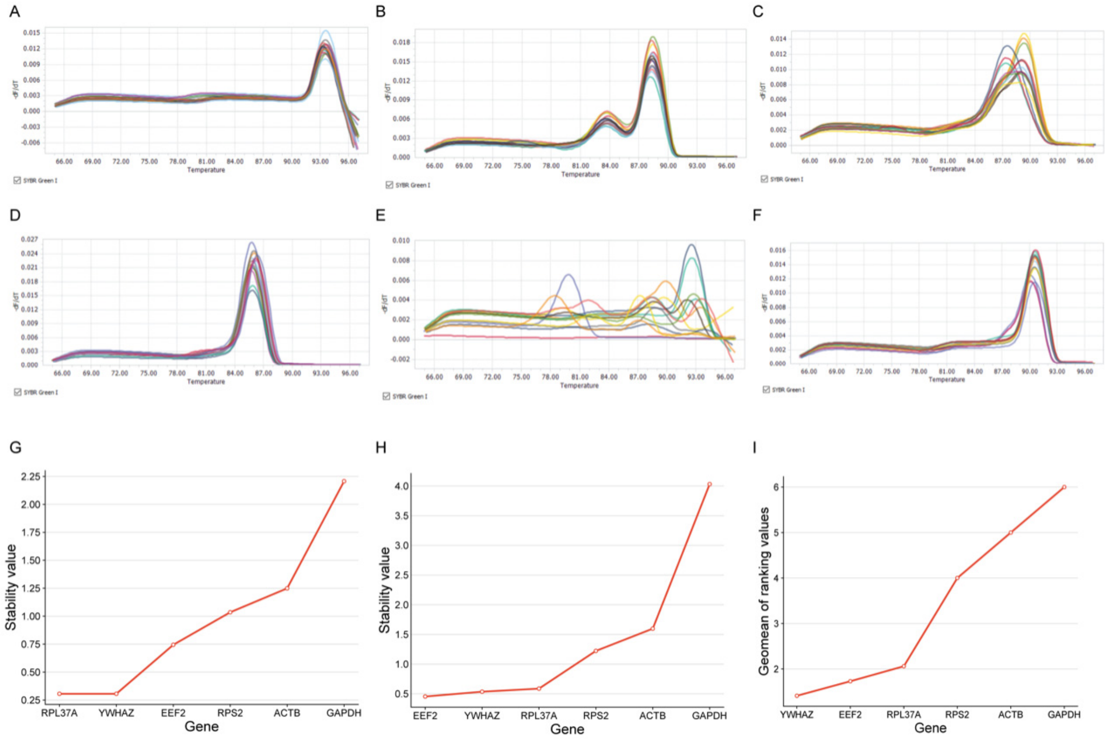


**Supplementary Figure S2 Validation of buffalo reference genes.** (A-F) Melting curve analysis of candidate reference genes; (G) Line plot of NormFinder analysis; (H) Line plot of geNorm analysis; (I) Line plot of RefFinder analysis

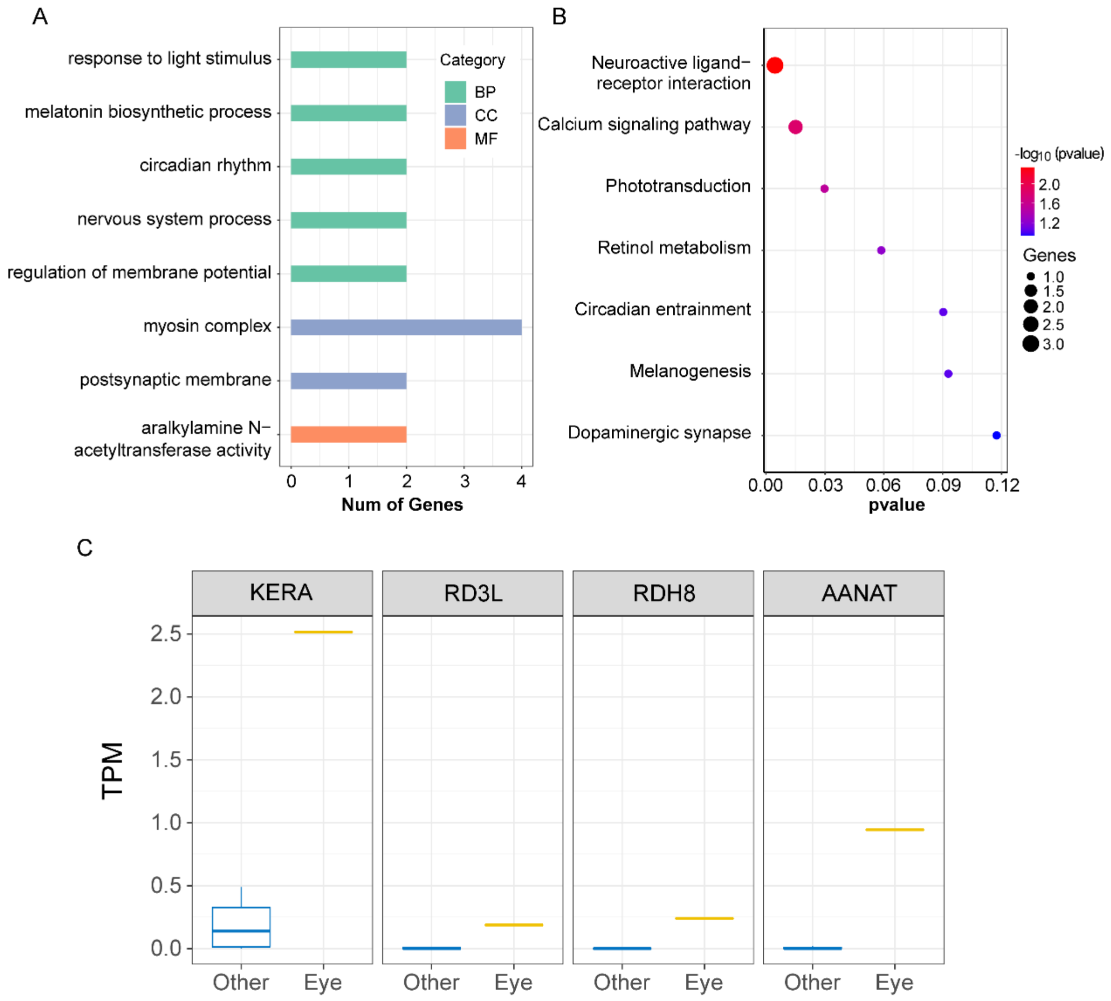


**Supplementary Figure S3 Analysis of eye TSGs.** (A) Bar plot of GO enrichment analysis for eyes TSGs; (B) Bubble plot of KEGG pathway enrichment analysis for eyes TSGs;(C) Heatmap of eyes TSGs expression

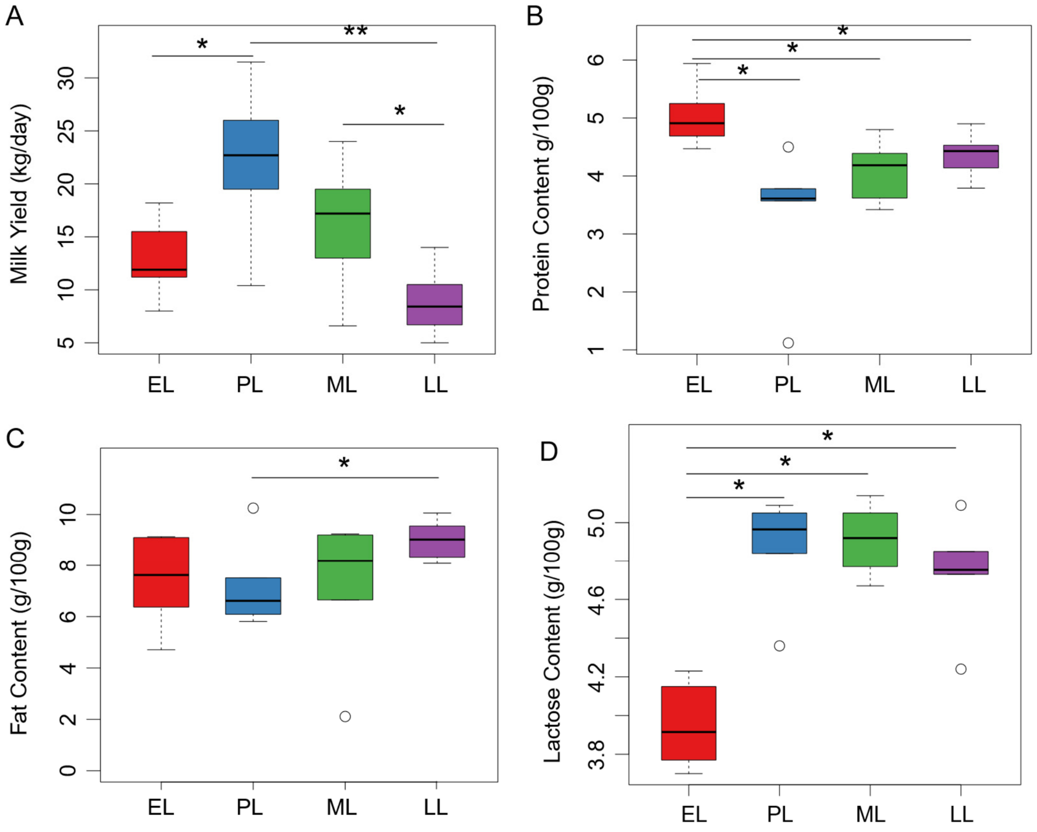


**Supplementary Figure S4 Analysis of milk yield and composition across lactation stages.** (A) Box plot of milk yield, * $p < 0.05$, ** $p < 0.01$; (B) Box plot of milk protein content; (C) Box plot of milk fat content; (D) Box plot of milk lactose content.

**Table S1 Table of filtering and quality control statistics for sequencing data of 20 tissues**

| sample | clean reads (M) | clean bases (G) | Q20 (%) | Q30 (%) | breed | Source |
|---|---|---|---|---|---|---|
| backfat1 | 59.13 | 8.82 | 97.18 | 92.63 | Murrah | Inhouse |
| backfat2 | 63.12 | 9.42 | 97.26 | 92.83 | Murrah | Inhouse |
| backfat3 | 55.09 | 8.17 | 97.08 | 92.49 | Nili-Ravi | Inhouse |
| backfat4 | 52.41 | 7.77 | 97.13 | 92.61 | Nili-Ravi | Inhouse |
| perirenalfat1 | 61.23 | 9.14 | 97.00 | 92.16 | Murrah | Inhouse |

| sample | clean reads (M) | clean bases (G) | Q20 (%) | Q30 (%) | breed | Source |
|---|---|---|---|---|---|---|
| perirenalfat2 | 69.43 | 10.34 | 97.04 | 92.31 | Murrah | Inhouse |
| perirenalfat4 | 59.35 | 8.85 | 96.98 | 92.14 | Nili-Ravi | Inhouse |
| cerebellum1 | 55.46 | 8.27 | 96.15 | 90.91 | Murrah | Inhouse |
| cerebellum2 | 66.24 | 9.81 | 97.49 | 93.18 | Murrah | Inhouse |
| cerebellum3 | 61.09 | 9.10 | 96.29 | 91.15 | Nili-Ravi | Inhouse |
| cerebrum1 | 58.58 | 8.73 | 96.22 | 91.07 | Murrah | Inhouse |
| cerebrum3 | 57.07 | 8.50 | 96.31 | 91.19 | Nili-Ravi | Inhouse |
| cerebrum4 | 79.87 | 11.83 | 97.18 | 92.67 | Nili-Ravi | Inhouse |
| duodenum1 | 51.38 | 7.62 | 97.52 | 93.31 | Murrah | Inhouse |
| duodenum3 | 59.24 | 8.82 | 96.37 | 91.38 | Nili-Ravi | Inhouse |
| duodenum4 | 50.49 | 7.51 | 97.15 | 92.67 | Nili-Ravi | Inhouse |
| heart2 | 68.90 | 10.18 | 97.53 | 93.25 | Murrah | Inhouse |
| heart3 | 80.24 | 11.67 | 97.50 | 93.48 | Nili-Ravi | Inhouse |
| heart4 | 62.54 | 9.32 | 96.47 | 91.55 | Nili-Ravi | Inhouse |
| hypothalamus1 | 68.40 | 10.15 | 97.46 | 93.14 | Murrah | Inhouse |
| hypothalamus2 | 57.60 | 8.53 | 97.50 | 93.29 | Murrah | Inhouse |
| jejunum1 | 40.27 | 4.75 | 98.28 | 96.49 | Mediterranea | PRJEB25226 |
| jejunum2 | 41.85 | 4.89 | 98.42 | 96.80 | Mediterranea | PRJEB25226 |
| jejunum3 | 40.41 | 4.74 | 97.64 | 95.10 | Mediterranea | PRJEB25226 |
| jejunum4 | 32.61 | 3.92 | 97.35 | 94.41 | Mediterranea | PRJEB25226 |
| kidney1 | 66.64 | 9.94 | 97.16 | 92.54 | Murrah | Inhouse |
| kidney2 | 71.79 | 10.64 | 97.40 | 92.99 | Murrah | Inhouse |
| kidney3 | 75.11 | 11.09 | 97.26 | 93.00 | Nili-Ravi | Inhouse |
| kidney4 | 55.37 | 8.21 | 97.20 | 92.76 | Nili-Ravi | Inhouse |
| liver1 | 60.89 | 9.08 | 96.28 | 91.17 | Murrah | Inhouse |
| liver2 | 61.12 | 9.12 | 96.51 | 91.70 | Murrah | Inhouse |
| liver3 | 50.81 | 7.59 | 96.43 | 91.46 | Nili-Ravi | Inhouse |
| liver4 | 59.82 | 8.93 | 96.39 | 91.35 | Nili-Ravi | Inhouse |
| lung1 | 58.88 | 8.77 | 96.89 | 92.09 | Murrah | Inhouse |

| sample | clean reads (M) | clean bases (G) | Q20 (%) | Q30 (%) | breed | Source |
|---|---|---|---|---|---|---|
| lung2 | 52.33 | 7.79 | 96.88 | 92.06 | Murrah | Inhouse |
| lung4 | 54.54 | 8.13 | 96.89 | 92.08 | Nili-Ravi | Inhouse |
| breast1 | 45.46 | 6.76 | 97.01 | 92.29 | Nili-Ravi | Inhouse |
| breast33 | 71.09 | 10.60 | 97.16 | 92.56 | Nili-Ravi | Inhouse |
| breast4 | 49.97 | 7.46 | 96.07 | 90.66 | Nili-Ravi | Inhouse |
| longissimus1 | 55.90 | 8.33 | 96.10 | 90.84 | Murrah | Inhouse |
| longissimus2 | 56.29 | 8.40 | 96.07 | 90.76 | Murrah | Inhouse |
| ovary2 | 181.67 | 21.14 | 97.84 | 95.39 | Mediterranea | PRJEB25226 |
| ovary3 | 220.01 | 25.99 | 98.06 | 96.09 | Mediterranea | PRJEB25226 |
| ovary5 | 188.26 | 21.97 | 97.83 | 95.73 | Mediterranea | PRJEB25226 |
| pituitarium1 | 65.48 | 9.76 | 96.59 | 91.71 | Murrah | Inhouse |
| pituitarium2 | 55.99 | 8.35 | 96.40 | 91.43 | Murrah | Inhouse |
| pituitarium3 | 68.97 | 10.26 | 96.77 | 92.14 | Nili-Ravi | Inhouse |
| pituitarium4 | 61.98 | 9.23 | 96.43 | 91.37 | Nili-Ravi | Inhouse |
| rectum1 | 71.42 | 10.57 | 97.11 | 92.62 | Murrah | Inhouse |
| rectum2 | 54.01 | 8.07 | 96.00 | 90.61 | Murrah | Inhouse |
| rectum3 | 54.29 | 8.10 | 96.31 | 91.23 | Nili-Ravi | Inhouse |
| rectum4 | 57.86 | 8.63 | 96.32 | 91.27 | Nili-Ravi | Inhouse |
| rumen1 | 60.65 | 9.02 | 97.25 | 92.94 | Murrah | Inhouse |
| rumen2 | 59.59 | 8.89 | 96.17 | 90.94 | Murrah | Inhouse |
| rumen4 | 44.92 | 5.34 | 97.74 | 95.22 | Mediterranea | PRJEB25226 |
| rumen6 | 41.93 | 4.96 | 97.72 | 95.22 | Mediterranea | PRJEB25226 |
| beipi1 | 52.34 | 7.78 | 97.01 | 92.31 | Murrah | Inhouse |
| beipi2 | 57.85 | 8.64 | 96.03 | 90.66 | Murrah | Inhouse |
| beipi3 | 58.40 | 8.64 | 97.03 | 92.34 | Nili-Ravi | Inhouse |
| spleen1 | 57.09 | 8.52 | 96.35 | 91.38 | Murrah | Inhouse |
| spleen2 | 62.91 | 9.38 | 97.11 | 92.46 | Murrah | Inhouse |
| spleen3 | 50.13 | 7.49 | 96.22 | 91.05 | Nili-Ravi | Inhouse |
| spleen4 | 50.26 | 7.51 | 96.06 | 90.72 | Nili-Ravi | Inhouse |

| sample | clean reads (M) | clean bases (G) | Q20 (%) | Q30 (%) | breed | Source |
|---|---|---|---|---|---|---|
| testis1 | 61.94 | 9.22 | 96.31 | 91.13 | Murrah | Inhouse |
| testis2 | 74.05 | 11.03 | 96.44 | 91.38 | Murrah | Inhouse |
| eye1 | 58.79 | 8.73 | 95.95 | 90.55 | Nili-Ravi | PRJNA1124431 |
| eye2 | 60.54 | 9.01 | 95.79 | 90.19 | Nili-Ravi | PRJNA1124431 |
| eye3 | 55.81 | 8.30 | 95.82 | 90.26 | Nili-Ravi | PRJNA1124431 |
| eye4 | 46.90 | 6.99 | 95.85 | 90.34 | Nili-Ravi | PRJNA1124431 |
| eye5 | 57.07 | 8.49 | 95.67 | 89.92 | Murrah | PRJNA1124431 |
| eye6 | 53.57 | 7.97 | 95.87 | 90.45 | Murrah | PRJNA1124431 |
| eye7 | 49.53 | 7.36 | 95.80 | 90.31 | Murrah | PRJNA1124431 |
| eye8 | 51.87 | 7.69 | 96.12 | 90.99 | Murrah | PRJNA1124431 |
| B7A* | 23.44 | 1.17 | 97.12 | 89.33 | Murrah | PRJNA480718 |
| B7B* | 23.04 | 1.15 | 96.79 | 88.65 | Murrah | PRJNA480718 |
| B50A* | 23.45 | 1.17 | 96.57 | 88.18 | Murrah | PRJNA480718 |
| B50B* | 23.09 | 1.15 | 96.83 | 88.62 | Murrah | PRJNA480718 |
| B140A* | 22.96 | 1.15 | 96.66 | 88.16 | Murrah | PRJNA480718 |
| B140B* | 22.56 | 1.13 | 97.07 | 89.01 | Murrah | PRJNA480718 |
| B280A* | 23.43 | 1.17 | 97.21 | 89.35 | Murrah | PRJNA480718 |
| B280B* | 23.37 | 1.17 | 96.57 | 87.82 | Murrah | PRJNA480718 |

*Mammary gland tissue samples were collected on day 7 (D7), 50 (D50), 140 (D140), and 280 (D280) after calving.

**Table S3 Multi-tissue marker gene table for buffalo**

| gene | tissue | gene | tissue | gene | tissue |
|---|---|---|---|---|---|
| GUCA1C | cerebrum | LOC102400620 | rumen | LOC102411110 | testis |
| LOC112579856 | cerebrum | LOC123465926 | skin | LOC112587646 | testis |
| LOC112581622 | cerebrum | LOC123464528 | skin | LOC123334475 | testis |
| LOC123334544 | cerebrum | LOC112583676 | skin | LOC112578420 | testis |
| SMIM28 | duodenum | LOC102390228 | skin | LOC123330530 | testis |
| LOC123333926 | eye | LOC123334226 | skin | LOC123329787 | testis |

| gene | tissue | gene | tissue | gene | tissue |
|---|---|---|---|---|---|
| LOC112587831 | heart | LOC102409514 | skin | LOC102409726 | testis |
| LOC102412758 | kidney | LOC102416247 | skin | LOC123465521 | testis |
| HELT | kidney | LOC123465669 | skin | LOC123330819 | testis |
| LOC112578416 | kidney | LOC123334230 | skin | LOC112580119 | testis |
| LOC123334155 | liver | LOC123330395 | skin | LOC123328492 | testis |
| LOC112581538 | liver | LOC102412164 | skin | LOC102395443 | testis |
| LOC112584579 | liver | LOC112586274 | skin | LOC123328095 | testis |
| LOC123333073 | liver | LOC102406135 | skin | LOC112578737 | testis |
| LOC112581919 | liver | LOC102395596 | skin | LOC123330972 | testis |
| LOC112586826 | liver | LOC112586256 | skin | LOC123331244 | testis |
| LOC112586185 | liver | LOC123332618 | skin | LOC123464975 | testis |
| LOC102414990 | lung | TCHHL1 | skin | LOC123465411 | testis |
| LALBA | mammary | LOC112580519 | skin | LOC102394425 | testis |
| LOC102391894 | mammary | LOC123330452 | skin | LOC123328399 | testis |
| LOC112584355 | mammary | CLPSL2 | skin | LOC123330041 | testis |
| LOC102399545 | mammary | LOC123327822 | skin | LOC102397723 | testis |
| LOC112578771 | mammary | LOC123328290 | spleen | LOC102411913 | testis |
| LOC123334466 | mammary | ASB17 | testis | LOC123464715 | testis |
| LOC102390352 | mammary | C23H10ORF120 | testis | LOC112582224 | testis |
| LOC102401752 | mammary | LOC112587909 | testis | LOC123465127 | testis |
| LOC102408611 | mammary | LOC123328940 | testis | LOC112580151 | testis |
| LOC112578211 | mammary | LOC102409886 | testis | LOC123464525 | testis |
| LOC102402666 | mammary | LY6K | testis | WFDC11 | testis |
| LOC102395554 | mammary | LOC112587110 | testis | LOC123332368 | testis |
| LOC102393704 | mammary | LOC112579845 | testis | LOC102414431 | testis |
| LOC123328916 | mammary | TERB2 | testis | LOC102409728 | testis |
| LOC102413724 | mammary | LOC123329168 | testis | LOC123465410 | testis |
| LOC112586295 | ovary | LOC112582346 | testis | LOC102408648 | testis |
| LOC112584701 | ovary | SMIM23 | testis | LOC102412335 | testis |
| LOC123331797 | ovary | LOC123465092 | testis | LOC102411252 | testis |
| LOC112585586 | ovary | PPP1R2C | testis | LOC123333065 | testis |
| LOC123331043 | ovary | LOC112579574 | testis | LOC102415317 | testis |
| LOC112581918 | ovary | LOC123329371 | testis | LOC102393498 | testis |
| LOC112580039 | ovary | LOC112578205 | testis | LOC123329830 | testis |
| LOC123333783 | ovary | LOC123332583 | testis | LOC123333310 | testis |
| LOC112584975 | ovary | LOC102394572 | testis | LOC123333771 | testis |
| LOC112583102 | ovary | LOC112579214 | testis | LOC123332366 | testis |
| LOC102403476 | ovary | LOC102415697 | testis | LOC112587515 | testis |
| LOC112587166 | ovary | LITAFD | testis | LOC123327965 | testis |
| LOC123331849 | ovary | LOC102409454 | testis | TEX28 | testis |
| LOC112587445 | ovary | LOC102392860 | testis | LOC123331882 | testis |
| LOC123334994 | ovary | TRIML1 | testis | LOC123333462 | testis |
| LOC112583038 | ovary | LOC112584421 | testis | LOC123328291 | testis |

| gene | tissue | gene | tissue | gene | tissue |
|---|---|---|---|---|---|
| LOC123334874 | ovary | LOC123465554 | testis | DEFB116 | testis |
| LOC112582435 | ovary | LOC123334426 | testis | LOC123465809 | testis |
| LOC123333208 | ovary | LOC123332697 | testis | LOC112580125 | testis |
| LOC112578570 | ovary | LOC123334969 | testis | LOC102403663 | testis |
| LOC123332474 | ovary | LOC102398518 | testis | LOC102405093 | testis |
| LOC112579259 | ovary | LOC112582352 | testis | LOC102389690 | testis |
| LOC112584178 | ovary | LOC112582192 | testis | LOC123334255 | testis |
| LOC123333347 | ovary | LOC102416621 | testis | LOC112586680 | testis |
| LOC112577906 | ovary | LOC123333591 | testis | LOC123331017 | testis |
| LOC112581310 | ovary | LOC123465894 | testis | LOC123328178 | testis |
| LOC112582999 | ovary | LOC112583981 | testis | LOC112581308 | testis |
| LOC123331151 | ovary | LOC112582263 | testis | LOC123465250 | testis |
| LOC123331732 | ovary | LOC112587418 | testis | FOXR2 | testis |
| LOC112581060 | ovary | LOC112582233 | testis | LOC123330271 | testis |
| LOC112581782 | ovary | LOC112582013 | testis | LOC123335116 | testis |
| LOC123332678 | ovary | LOC112582217 | testis | LOC112580141 | testis |
| LOC112582459 | ovary | LOC123334856 | testis | LOC112580117 | testis |
| LOC112586932 | ovary | LOC123331471 | testis | LOC123329387 | testis |
| LOC112580025 | ovary | LOC102406655 | testis | LOC123332815 | testis |
| LOC112580005 | ovary | LOC102391442 | testis | LOC102390884 | testis |
| LOC123332688 | ovary | LOC112585773 | testis | LOC123335027 | testis |
| LOC112584817 | ovary | LOC102389223 | testis | LOC102393675 | testis |
| LOC123334141 | ovary | LOC102390668 | testis | LOC102412988 | testis |
| LOC112584781 | ovary | LOC112582126 | testis | LOC102390396 | testis |
| LOC123328265 | ovary | LOC123333931 | testis | LOC102395666 | testis |
| LOC123330705 | ovary | LOC123331848 | testis | LOC123331897 | testis |
| LOC123334457 | ovary | LOC102416293 | testis | LOC123329587 | testis |
| LOC112582422 | ovary | LOC123331440 | testis | LOC123332768 | testis |
| LOC102394312 | ovary | LOC102397281 | testis | LOC123334413 | testis |
| LOC112587180 | ovary | LOC102413820 | testis | LOC102408527 | testis |
| LOC112578578 | ovary | LOC112579407 | testis | LOC112585489 | testis |
| LOC123335100 | ovary | LOC112577799 | testis | LOC112582364 | testis |
| LOC112580031 | ovary | LOC123327768 | testis | LOC102415702 | testis |
| LOC123329297 | ovary | LOC112585135 | testis | LOC123465088 | testis |
| LOC123330066 | ovary | LOC123327536 | testis | LOC102414516 | testis |
| LOC112579580 | pituitarium | LOC123332592 | testis | LOC123330051 | testis |
| LOC102414080 | pituitarium | LOC102406457 | testis | LOC112578984 | testis |
| LOC123329241 | pituitarium | LOC123328116 | testis | LOC102414573 | testis |
| LOC123331037 | pituitarium | LOC102407446 | testis | LOC102410180 | testis |
| HOXD12 | rectum | LOC102415969 | testis | LOC102414276 | testis |

**Table S5 Primer sequences for housekeeping genes**

| Primer | Sequence |
|---|---|
| ACTB-F | GATGATGATATTGCCGCGCTC |
| ACTB-R | ACCATTACGCCCTGGTGC |
| GAPDH-F | TGATGGGTGTGAACCACGAG |
| GAPDH-R | CGTGGACGGTGGTCATAAGT |
| YWHAZ-F | TAGCCTGTGAGCAGCGAGAT |
| YWHAZ-R | CATGACTGGATGTTCTGTGTCC |
| RPS2-F | GCGCCTTATAAAGACACCGTC |
| RPS2-R | GTAACGGGGAGCCACTCCT |
| EEF2-F | GTGGGGAGACCGGTACTTTG |
| EEF2-R | TCAGAAGTGGTTTGCCCTCC |
| RPL37A-F | ATGGTGCCTCCCTCAGGAAAAT |
| RPL37A-R | TTGTAGGTCCAGGCACCACCA |
